# Natural antibody IgG levels are associated with HBeAg-positivity and seroconversion in chronic hepatitis B patients treated with entecavir

**DOI:** 10.1038/s41598-022-08457-w

**Published:** 2022-03-14

**Authors:** Youkyung H. Choi, Hyun Woong Lee, Michael A. Purdy

**Affiliations:** 1 Laboratory Branch, Division of Viral Hepatitis, National Center for HIV, Viral Hepatitis, STD and TB Prevention (NCHHSTP), US Centers for Disease Control and Prevention (CDC), Atlanta, GA 30329 USA; 2 Department of Internal Medicine, Gangnam Severance Hospital, Yonsei University College of Medicine, Seoul, South Korea

**Keywords:** Microbiology, Virology, Hepatitis B virus

## Abstract

B1 cell-derived natural antibodies are non-specific polyreactive antibodies and can activate the complement pathway leading to lysis of enveloped virus particles before activation of the adaptive immune response. We investigated the relationship between natural antibody levels and treatment outcomes of 126 treatment-naïve chronic hepatitis B (CHB) patients, who underwent entecavir (ETV) treatment. Serum IgG1-3 and complement C3 levels were significantly higher in HBeAg-positive patients. In pre-treatment, IgG1 (odd ratios [OR] 2.3, p < 0.0001), IgG2 (OR 9.8, p < 0.0001), IgG3 (OR 7.4, p < 0.0001), and C3 (OR 7.2, p < 0.0001) were associated with HBeAg-positive patients. At baseline, IgG2 (OR 10.2, p = 0.025), IgG4, (OR 3.4, p = 0.026), and complement C1q (OR 5.0, p = 0.0068) were associated with seroconverters. Post-treatment levels of IgG1-4 and C3/C1q were also associated with HBeAg-positive patients and seroconverters. High levels of IgG2-4 and C1q were observed in seroconverters but not in virological responders. Thus, high pretreatment and post-treatment levels of natural antibody IgG1-4, complement C3, and/or C1q were significantly associated with HBeAg-positivity and HBeAg seroconverters in CHB patients with ETV treatment. These results suggest that the presence of preexisting host immunity against chronic hepatitis B is closely related to outcome of ETV treatment.

## Introduction

Hepatitis B virus (HBV) infection causes liver diseases, which affected an estimated 257 million persons globally in 2015^1,2^. There were about 1.89 million persons with chronic hepatitis B (CHB) in the United States in 2018^3,4^. The course of hepatitis B can be asymptomatic or variable including symptoms like nausea, vomiting, diarrhea, anorexia, jaundice, and headaches^5^. Hepatitis B surface antigen (HBsAg) can be detected in the serum from several weeks before onset of symptoms to months after onset^6^ and is present in the serum during acute infection and persists in chronic phase of infections^7^. The presence of HBsAg indicates that the person is potentially infectious. Resolved CHB infection is defined by clearance of HBsAg with acquisition of antibody to HBsAg^8^. Most adult patients recover completely from their HBV infection, but about 5 to 10% progress to become asymptomatic carriers or develop chronic hepatitis, potentially resulting in cirrhosis and/or liver cancer^9,10^.

The current treatment of adults with CHB includes nucleotide analogues (NA) inhibiting the reverse transcription of pregenomic RNA to HBV DNA^8^. Success of anti-viral therapy against CHB is associated with normalization of alanine aminotransferase (ALT) activity, loss of HBsAg with or without detection of antibody to HBsAg (anti-HBs), and improvement in liver histology^8,11^. However, functional cure is only achieved in 1–5% of patients with more than 10 years of NA therapy^8,12,13^.

Antigen-specific antibodies produced by viral infection or vaccines are effective at preventing viral disease. However, humans and higher primates possess “natural antibodies” (IgA, IgM, and IgG) which are present in the serum before any viral infections^14,15^. B1 cell-derived natural antibody helps to dispose of cells that die or trigger opsonization of the invading pathogen and invoke activation of immune pathways leading to lysis of enveloped virus particles long before the adaptive immune response is activated^16–18^. In addition, complement facilitates the uptake and destruction of pathogens by phagocytic cells^19^. Innate B-cell responses are active during the immune active phase in HBeAg-positive patients with chronic hepatitis^20,21^. IgG is the most common antibody (70% to 80%) and has four subtypes (IgG1, IgG2, IgG3, and IgG4)^22^. High levels of IgG1 and IgG3 in convalescent serum from patients recovering from HBV infection and IgG4 were found in circulating immune complexes from patients with CHB^23^. A high level of serum fucosyl-agalactosyl IgG1 present before NA treatment was associated with liver inflammatory severity and damage and favors the treatment outcome for HBeAg-positive CHB^24^. Other than HBV, natural antibody IgM and the classical complement pathway work to neutralize influenza virus in the absence of prior immunity^17^.

We studied the association between the serum natural antibody levels and outcomes of long-term NA treatment in treatment naïve CHB patients. In this study, serum levels of natural antibodies separated on different treatment outcomes were assessed in pre- and post-treatment serum samples of patients, who underwent entecavir (ETV) treatment. The serum levels of IgG isotypes (IgG1, IgG2, IgG3, and IgG4), complement C3 and C1q, and inflammatory cytokines were compared in HBeAg-positive and HBeAg-negative patients before treatment and patients with virological response (VR), partial virological response (PVR), seroconverters (SC), and non-SC post-treatment.

## Results

### Baseline patient characteristics

**Figure 1:**
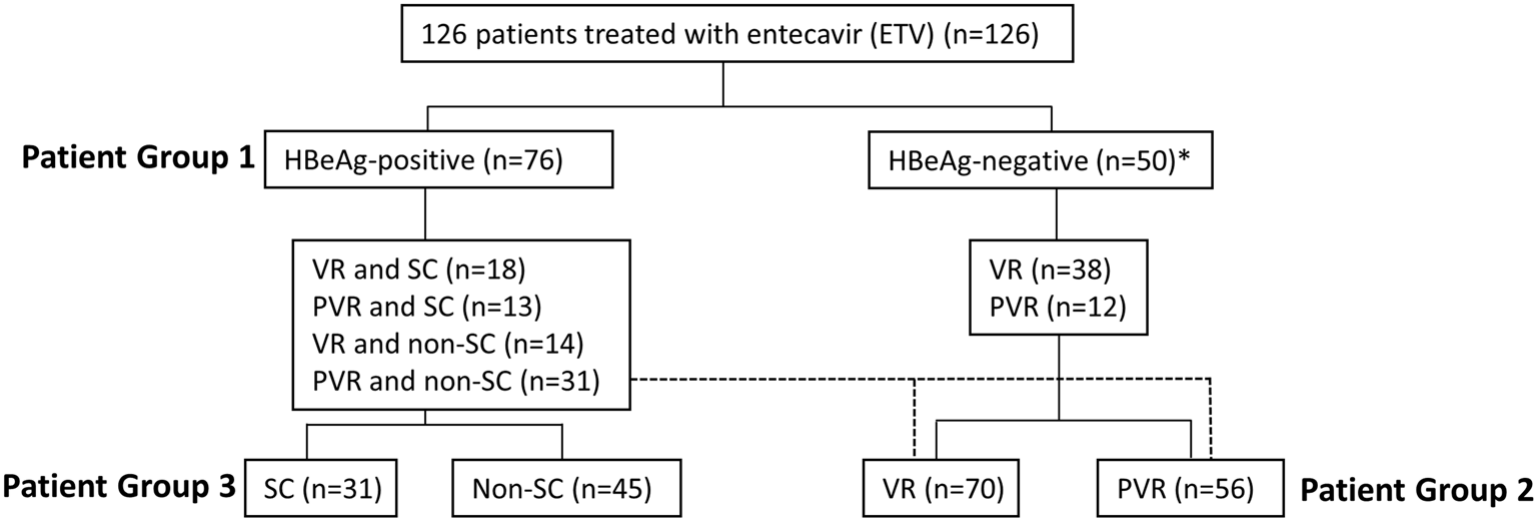
Treatment-naïve chronic hepatitis B patients treated with entecavir (ETV) and treatment outcome. All patients (n = 126) were treated with entecavir for 22–87 months. Three patient groups were used, HBeAg-positive vs. HBeAg-negative; VR, virological response vs. PVR; and SC, seroconverters vs non-SC, no seroconversion. Asterisk, HBeAg-negative patients were anti-HBe antibody positive before the treatment.

Seventy-six (60%) of 126 patients were HBeAg-positive and 50 (40%) were HBeAg-negative (Fig. 1).

The mean age of the HBeAg-positive patients was 52 years and 56 years for the HBeAg-negative patients. The HBeAg-positive patients were younger than the HBeAg-negative patients, but this difference was not statistically significant (*p* = 0.147) (Table 1). Both HBeAg-positive and HBeAg-negative patients had more males than females. For HBeAg-positive patients, the mean baseline ALT level was 167 IU/L, and serum HBV DNA was 9.2 log_10_ copies/ml. The mean baseline ALT level was 119 IU/L, and serum HBV DNA was 7.9 log_10_ copies/ml in the HBeAg-negative patients. The ALT levels and HBV DNA levels were higher in the HBeAg-positive patients than the HBeAg-negative patients. The mean duration of ETV therapy was 64 months for HBeAg-positive patients and 62 months for the HBeAg-negative patients.

**Table 1:** Baseline clinical characteristics of 126 patients with chronic hepatitis B.

	HBeAg-positive	HBeAg-negative	*P* value
No. patients (%)	76 (60)	50 (40)	
Mean age (years)	52 (24–88)	56 (33–75)	0.147
Male/female (no, %)	63/13 (83/17)	29/21 (58/42)	< 0.001
Baseline ALT (IU/L)	167 (23–400)	119 (11–340)	0.013
Baseline HBV DNA titer (log_10_ copies/ml)	9.2 (4.6–10.6)	7.9 (4.7–9.6)	0.017
Mean treatment duration (mo)	64 (24–86)	62 (22–87)	0.148

### Clinical outcomes in treatment naïve CHB patients after ETV monotherapy

Seventy (56%) of 126 treatment-naïve patients were virological responders (VR) defined as undetectable HBV DNA at 1 year after ETV treatment and 56 (44%) patients were partial virological responders (PVR) defined as a decrease in HBV DNA of more than 1 log_10_ copies/ml but detectable HBV DNA at 1 year of therapy (Fig. 1 and Table 2).

**Table 2:** Clinical characteristics of treatment outcome of CHB patients after ETV monotherapy.

	Viral response (n = 70)	Partial viral response (n = 56)	p value	HBeAg SC (n = 31)	Non-HBeAg SC (n = 45)	p value
Mean age (years)	53 (33–75)	53 (24–88)	0.442	59 (34–88)	59 (24–75)	0.323
Male/female (no, %)	48/20 (71/29)	42/14 (75/25)	0.357	26/5 (34/6.6)	37/8 (49/11)	0.393
Baseline ALT (IU/L) (range)	156 (16–400)	138 (11–396)	0.368	180 (32–396)	157 (23–400)	0.396
HBeAg positive (no, %)	31 (41)	45 (59)	0.0016	n/a	n/a	
Virological response at 1 year (no, %)	n/a	n/a		18 (58)	14 (31)	0.063
Baseline HBV DNA (log10 copies/ml)	9.0 (4.6–10.6)	9.0 (5.3–9.8)	0.37	8.8 (4.6–9.6)	9.3 (5.6–10.6)	0.07
Mean treatment duration (mo)	62 (22–84)	64 (27–87)	0.111	66(24–86)	63 (27–84)	0.393

*SC* seroconversion.

The percentage of VR at 1 year after ETV treatment was higher in HBeAg-negative patients (59%) than in the HBeAg-positive patients (41%) patients. After the ETV therapy, 31 (41%) of 76 HBeAg-positive patients were HBeAg negative and developed anti-HBeAg antibody indicating seroconversion (SC) and 45 (59%) patients did not lose HBeAg or develop an anti-HBeAg antibody response, indicating a lack of seroconversion (non-SC). The VR/PVR and SC/non-SC groups showed similar clinical characteristics with regards to mean age, ratio between male and female, baseline HBV DNA levels, and mean months of treatment duration (Table 2). Mean baseline ALT levels were higher in the VR patients and SC patients than PVR and non-SC patients, respectively. Patients with non-SC had higher baseline HBV DNA levels. However, ALT levels and HBV DNA titers were not statistically significant.

The treatment outcomes after ETV therapy were divided into three patient comparison groups: HBeAg-positive vs. HBeAg-negative patients before ETV treatment (Patient Group 1), VR vs. PVR (Patient Group 2), and SC vs. non-SC after ETV treatment (Patient Group 3) (Fig. 1). Serum levels of IgG subtypes (IgG1-4), C3, C1q, and IL-2, IL-8, IL-6, IL-10, IFN-α, IFN-γ, TNF-α, granzyme, and TRAIL were evaluated and compared across these groups.

### Levels of natural antibody subtypes (IgG1–4) and complement C3 and C1q in HBeAg-positive and HBeAg-negative patients

**Figure 2:**
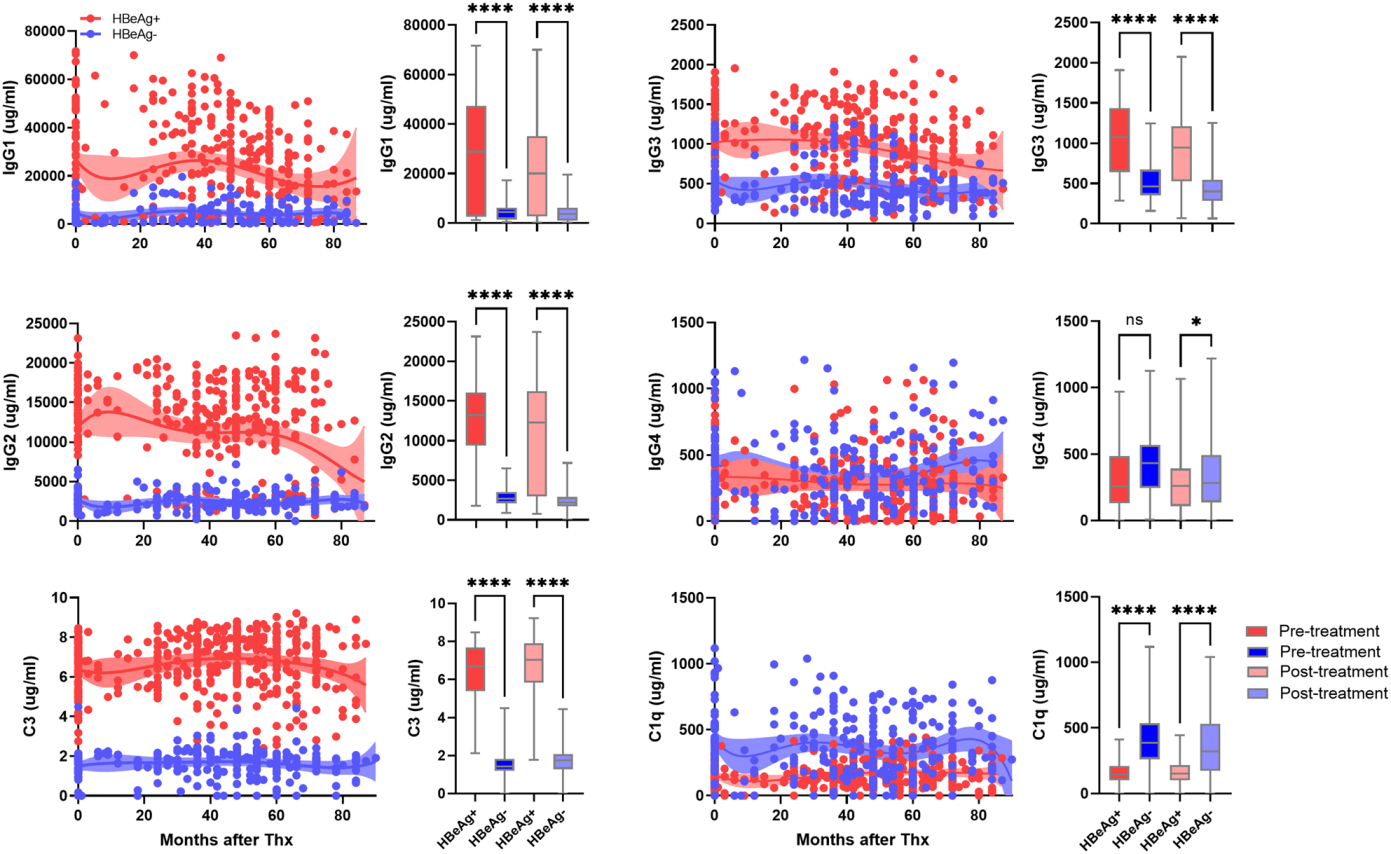
Serum levels of IgG subtypes and complement C3 and C1q in CHB patients with HBeAg-positive and HBeAg-negative patients. Serum levels of IgG1-4, complement C3, and C1q were measured in pretreatment and posttreatment (1–87 months) serum samples of HBeAg-positive (n = 76, red dots) and HBeAg-negative patients (n = 50, blue dots). Shaded areas indicate 95% confidence intervals around the regression lines. Bar graphs represent concentration of IgG1-4, complement C3, and C1q in pre-treatment (dark red), post-treatment (light red) of HBeAg-positive and pre-treatment (dark blue), post-treatment (light blue) of HBeAg-negative patients. *p ≤ 0.05, **p ≤ 0.01, ***p ≤ 0.001, ****p ≤ 0.0001.

Levels of natural antibody subtypes (IgG1–4) and complement C3 and C1q were analyzed before (pre-treatment, 0 month) and after ETV therapy (post-treatment). IgG1–3 and C3 levels were significantly higher in both pre-treatment and post-treatment of HBeAg-positive patients compared to that of HBeAg-negative patients (Fig. 2). IgG4 and complement C1q levels were higher in pre-treatment and post-treatment of HBeAg-negative patients compared to HBeAg-positive patients. Levels of IgG1-4 and complement C3 and C1q in 24 normal human serum samples (n = 24) were analyzed as control (IgG1, 2824 ± 776 µg/ml; IgG2, 846 ± 448 µg/ml; IgG3, 422 ± 164 µg/ml; IgG1, 178 ± 139 µg/ml; C3, 2.7 ± 0.21 µg/ml; C1q, 44 ± 8.4 µg/ml).

### Levels of natural antibody subtypes (IgG1–4) and complement C3 and C1q in patients with VR, PVR, SC, and non-SC

**Figure 3:**
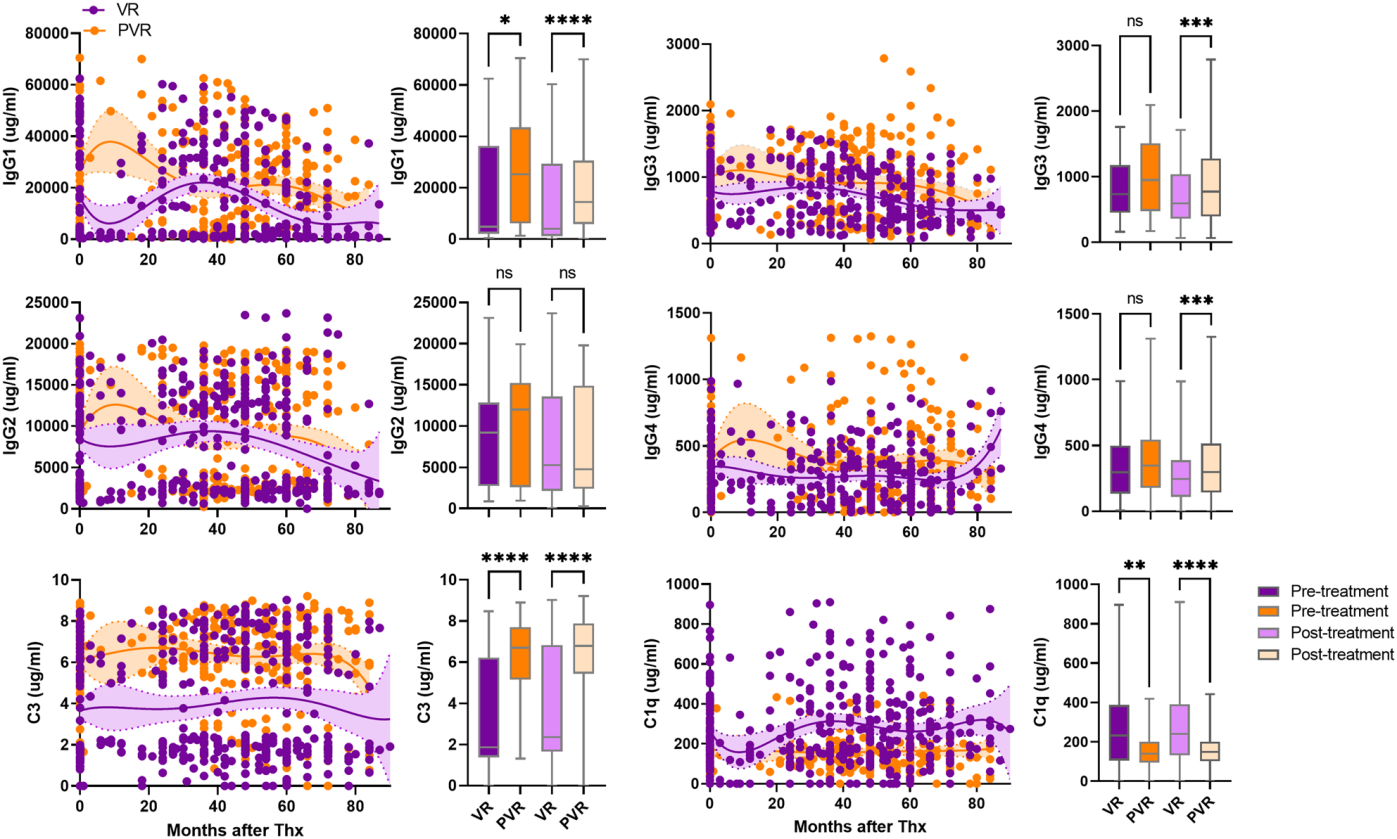
Levels of IgG subtypes and complement C3 and C1q in serum of CHB patients with virological response (VR) and partial virological response (PVR) patients. Serum levels of IgG1–4, complement C3, and C1q were measured in VR patients (n = 70, purple dots) and PVR patients (n = 56, orange dots). Shaded areas indicate 95% confidence intervals around the regression lines. Bar graphs represent concentration of IgG1-4, complement C3 and C1q in pre-treatment (dark purple), post-treatment (light purple) of VR and pre-treatment (dark orange), post-treatment (light orange) of PVR patients. *p ≤ 0.05, **p ≤ 0.01, ***p ≤ 0.001, ****p ≤ 0.0001.

**Figure 4:**
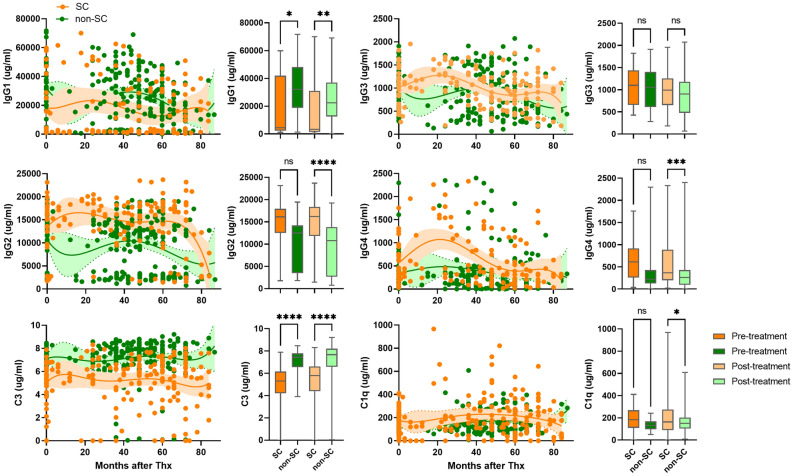
Levels of IgG subtypes and complement in serum of CHB patients with loss of HBeAg and detection of anti-HBe antibody, seroconversion (SC) and non-SC. Serum levels of IgG1–4, complement C3, and C1q were measured in SC patients (n = 31, orange dots) and non-SC patients (n = 45, green dots). Shaded areas indicate 95% confidence intervals around the regression lines. Bar graphs represent concentration of IgG1–4, complement C3 and C1q in pre-treatment (dark orange), post-treatment (light orange) of SC and pre-treatment (dark green), post-treatment (light green) of non-SC patients. *p ≤ 0.05, **p ≤ 0.01, ***p ≤ 0.001, ****p ≤ 0.0001.

Pre-treatment levels of IgG1–4 and C3 were higher in the PVR patients than in the VR patients, (Fig. 3). However, changes in levels of IgG2 and IgG3 levels were not statistically significant. Pre- and post-treatment C1q levels were higher in the VR patients than in the PVR patients. Post-treatment levels of IgG1, IgG3 and C3 were also higher in the PVR patients compared to those in the VR patients, but IgG3 between PVR and VR patients were not statistically significant. Pre-treatment levels of IgG1 and C3 were higher in the non-SC patients than in the SC patients (Fig. 4). Post-treatment IgG2, IgG3, IgG4, and C1q were higher in the SC patients than those in the non-SC patient. Post-treatment levels of IgG1 and C3 in the non-SC patients were higher than in the SC patients.

### Cytokine levels in patients with HBeAg-positive, HBeAg-negative, VR, PVR, SC, and non-SC

**Figure 5:**
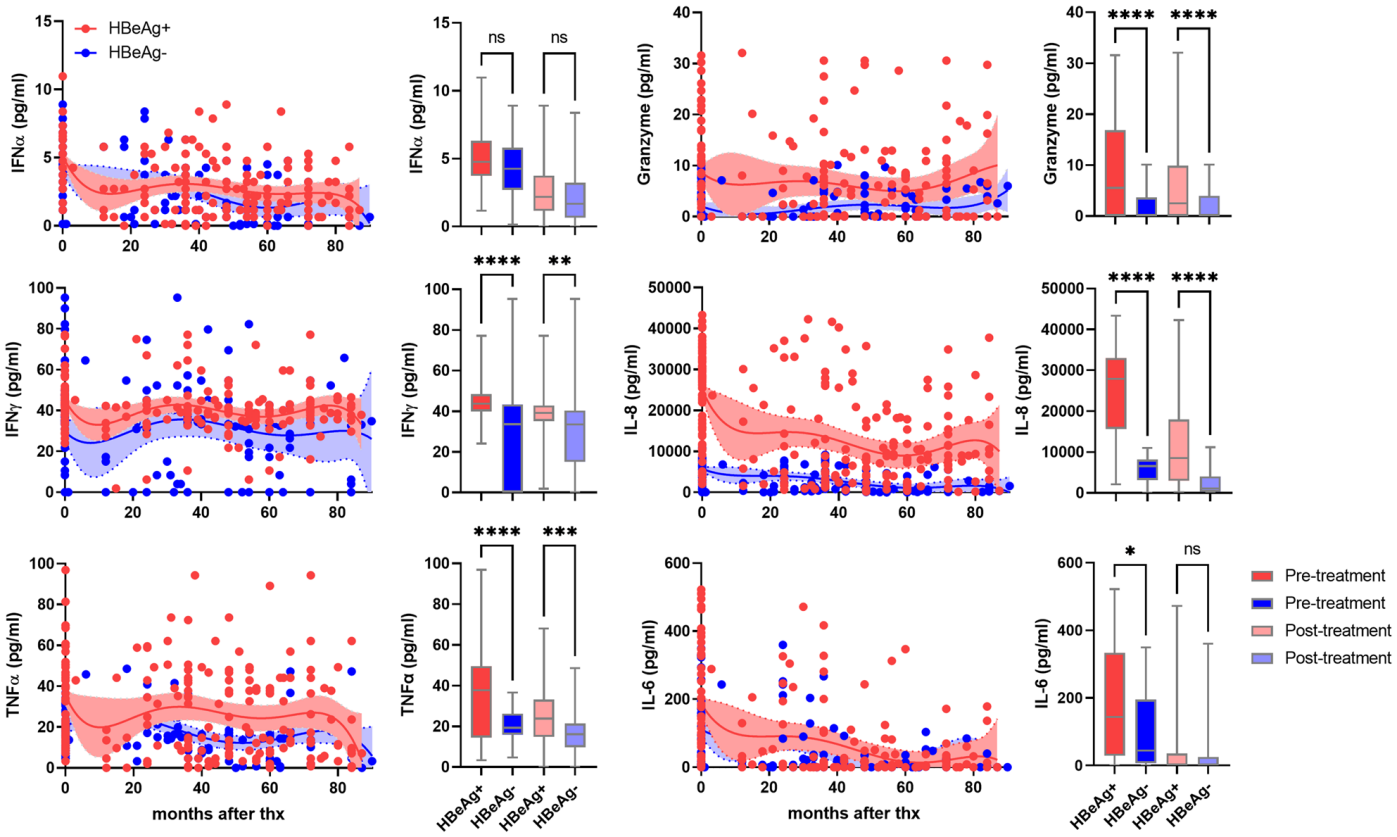
Cytokine levels in HBeAg-positive patients and HBeAg-negative patients. Circulating IFNα, IFNγ, TNFα, granzyme, IL-8, and IL-6 levels in HBeAg-positive patients (red dots) and HBeAg-negative patients (blue dots). Shaded areas indicate 95% confidence intervals around the regression lines. Bar graphs represent concentration of each cytokine in pre-treatment (dark red), post-treatment (light red) of HBeAg-positive and pre-treatment (dark blue), post-treatment (light blue) of HBeAg-negative patients. *p ≤ 0.05, **p ≤ 0.01, ***p ≤ 0.001, ****p ≤ 0.0001.

**Figure 6:**
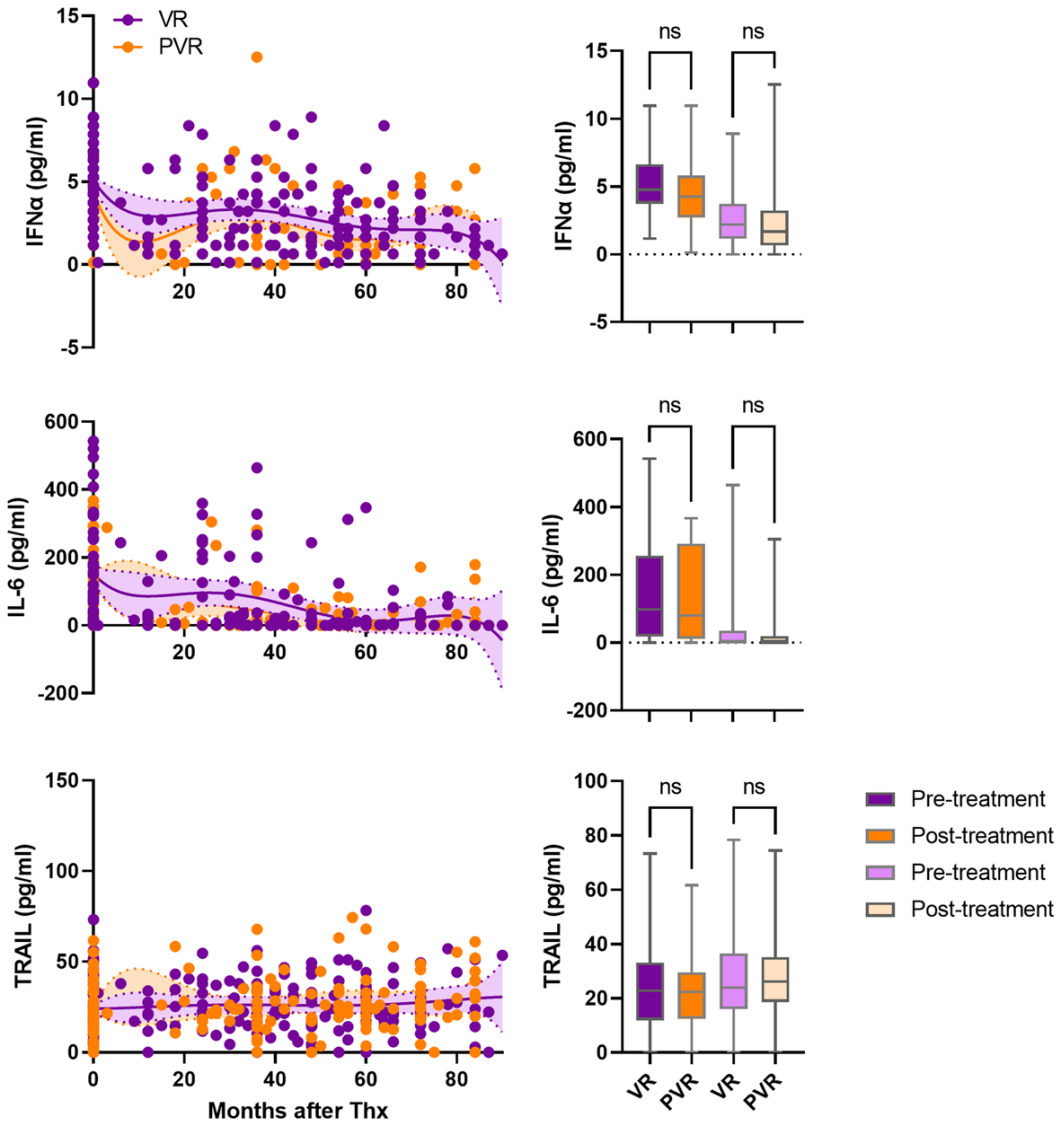
Levels of cytokine levels in VR patients and PVR patients. Circulating IFNα, IL-6, and TRAIL levels in VR (purple dots) patients and PVR patients (orange dots). Shaded areas indicate 95% confidence intervals around the regression lines. Bar graphs represent concentration of each cytokine in pre-treatment (dark purple), post-treatment (light purple) of VR and pre-treatment (dark orange), post-treatment (light orange) of PVR patients. *p ≤ 0.05, **p ≤ 0.01, ***p ≤ 0.001, ****p ≤ 0.0001.

Pretreatment serum levels of IFNγ, TNFα, granzyme, IL-8, and IL-6 were higher in HBeAg-positive patients (Fig. 5). Post-treatment levels of, IFNγ, TNFα, granzyme, IL-8 in the HBeAg-positive patients were higher than in the HBeAg-negative patients. Post-treatment levels of IL-10 and TRAIL were higher in HBeAg negative and HBeAg positive patients, respectively (Fig. S1). IFNα, IL-6, and TRAIL levels in pre- and post-treatment between VR and PVR were not statistically significant (Fig. 6). Pretreatment levels of IL-8, IL-10, TNFα, and granzyme were higher in the PVR patients than those in the VR patients (Fig. S2).

**Figure 7:**
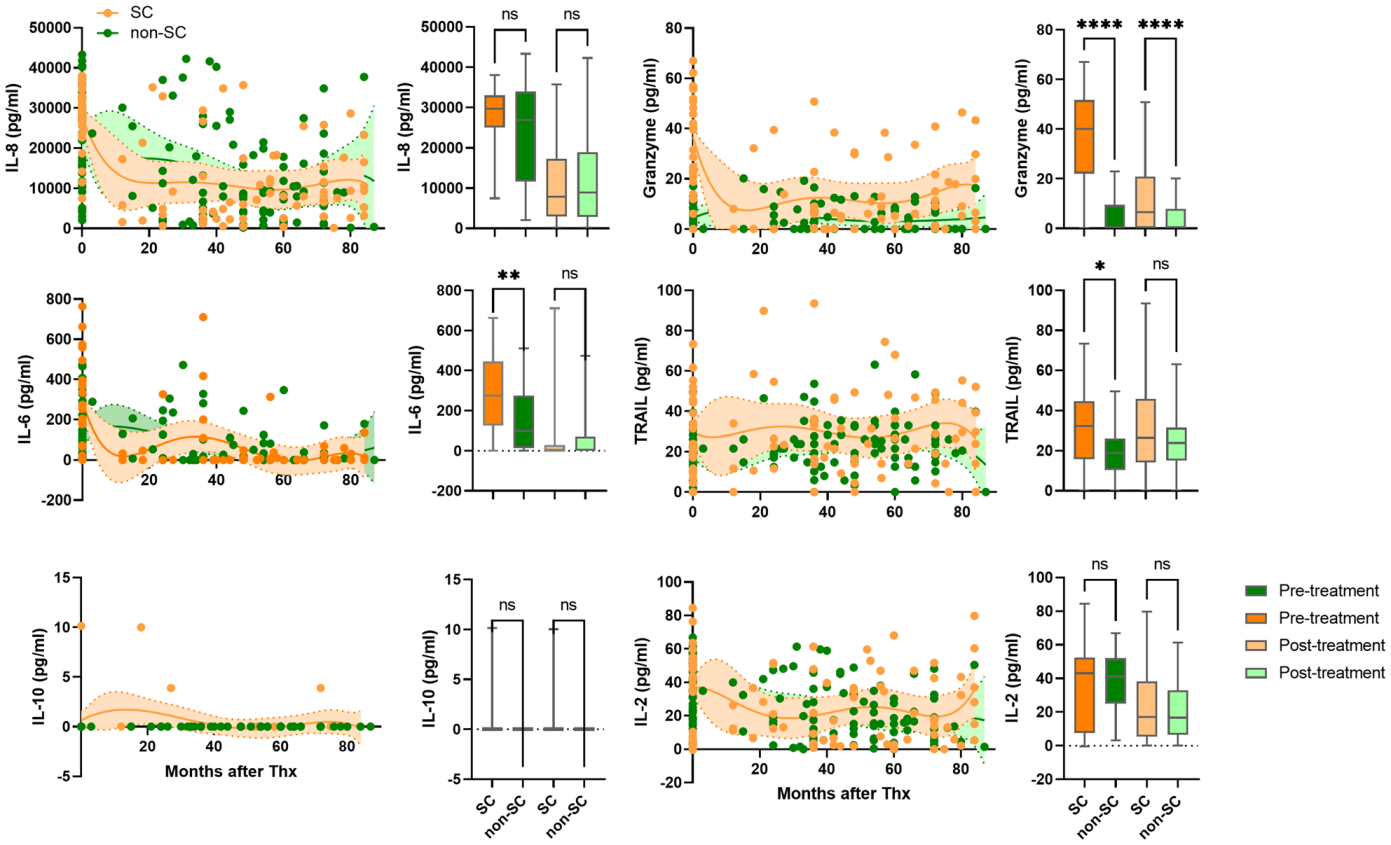
Levels of cytokine levels in SC patients and non-SC patients. Circulating IL-8, IL-6, IL-10, granzyme, TRAIL, and IL-2 in SC patients (orange dots) and non-SC patients (green dots). Shaded areas indicate 95% confidence intervals around the regression lines. Bar graphs represent concentration of each cytokine in in pre-treatment (dark orange), post-treatment (light orange) of SC and pre-treatment (dark green), post-treatment (light green) of non-SC patients. *p ≤ 0.05, **p ≤ 0.01, ***p ≤ 0.001, ****p ≤ 0.0001.

Pre-treatment, IL-6, granzyme, and TRAIL levels were significantly higher in the SC than in non-SC (Fig. 7). Post-treatment IFNα, IFNγ, and TNFα were higher in the non-SC patients compared to the SC patients (Fig. S3). However, IFNγ levels were not statistically significant. Post-treatment levels of granzyme in the SC patients and IFNα and TNFα in the non-SC patients were significantly higher compared to those in the non-SC patients and the SC patients, respectively.

### Odd ratios of natural antibody, complement, and inflammatory cytokines in three patient groups

**Figure 8:**
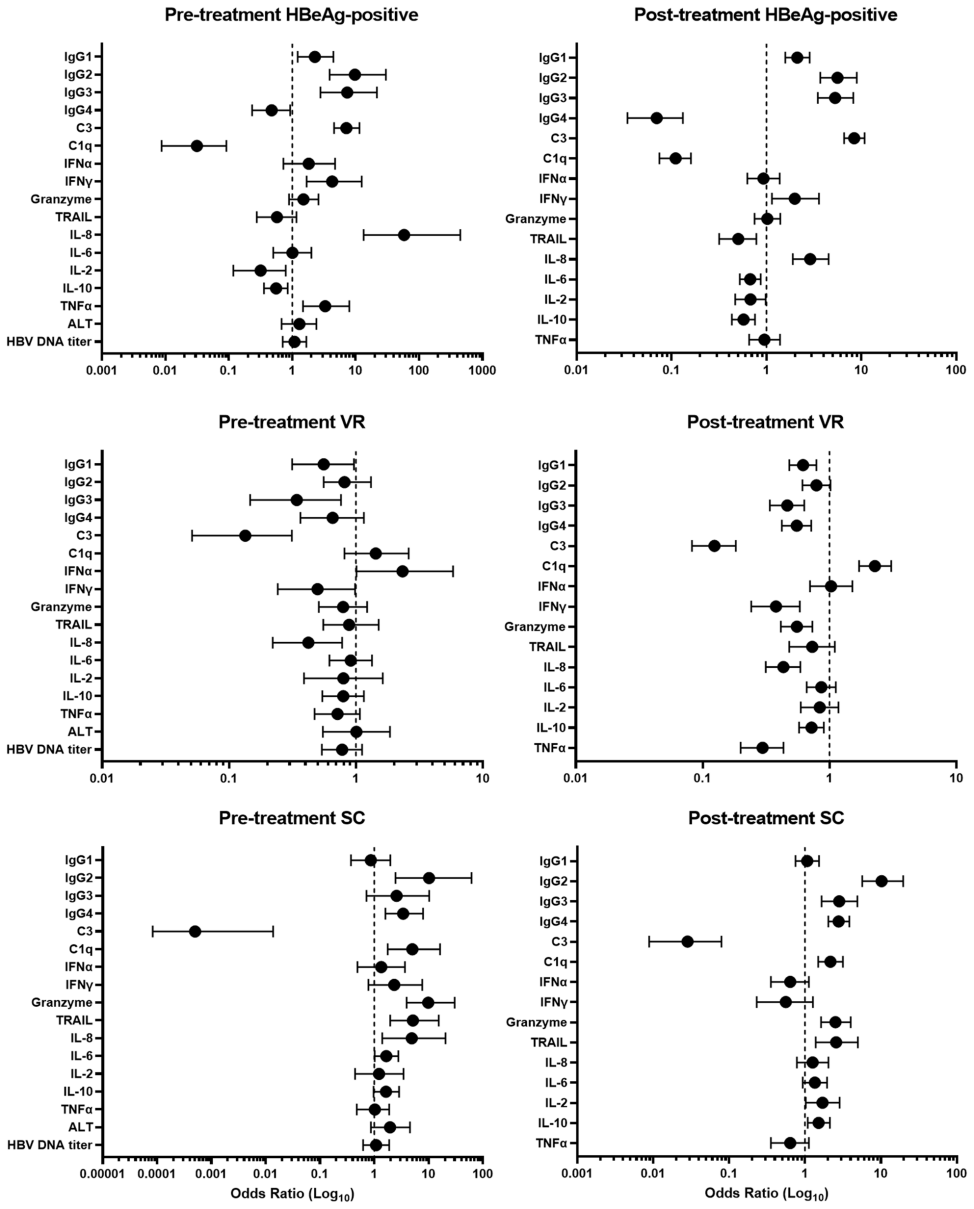
Association between IgG1–4, C3, C1q, cytokines and CHB patients with different treatment outcomes. **(A)** Forest plot of pre-treatment (baseline) levels of IgG1–4, C3, C1q, and cytokines in HBeAg-positve vs. HBeAg-negative; VR vs. PVR; SC vs. non-SC. **(B)** Post-treatment levels of the analytes in HBeAg-positve vs. HBeAg-negative; VR vs. PVR; SC vs. non-SC. Odd ratios were calculated using simple logistic regression models fitted with the outcome variables. The horizontal lines of each variable represent the 95% confidence interval of each odd ratio.

The association between natural antibodies, complement, inflammatory cytokines, and the treatment outcome are shown as forest plots (Fig. 8). IgG1–4, C3 or C1q were associated with HBeAg-positive and seroconversion patients in both pre-treatment (baseline) and posttreatment serum samples. However, IgG1–4 levels were not associated with the VR patients in pre- and post-treatment samples except for C1q [OR 2.3 (95% CI = 1.7–3.1), *p* < 0.0001 in post-treatment].

In pre-treatment samples, IgG1 (OR 2.3 [95%CI 1.2–4.5], *p* < 0.0001), IgG2 (OR 9.8 [95%CI 3.4–29.98], *p* < 0.0001), IgG3 (OR 7.4 [95%CI 2.8–21.7], *p* < 0.0001), and C3 (OR 7.2 [95%CI 4.6–11.5], *p* < 0.0001) were closely associated with HBeAg-positive patients and post-treatment serum samples (IgG1, OR 2.1 [95%CI 1.6–2.7], *p* < 0.0001), IgG2, OR 6.0 [95%CI 4.1–9.0], *p* < 0.0001), IgG3 (OR 5.5 [95%CI 3.7–8.2], *p* < 0.0001), and C3 (OR 8.4 [95%CI 6.7–10.8], *p* < 0.0001). High levels of IgG2-4 and C1q levels were associated with the SC patients in pre-treatment serum samples (IgG2, OR 10.2 [95% CI = 2.46–61.91], *p* = 0.025; IgG3, OR 2.6 [95% CI = 0.7–10.24], *p* = 0.57; IgG4, OR 3.4 [95% CI = 1.6–7.9], *p* = 0.026; C1q, OR 5.0 [95% CI = 1.8–16.22], *p* = 0.0068) and post-treatment samples (IgG2, OR 10.2 [95% CI = 5.7–19.63], *p* < 0.0001; IgG3, OR 2.8 [95% CI = 1.7–4.9], *p* = 0.083; IgG4, OR 2.8 [95% CI = 2.1–3.8], *p* = 0.0001; C1q, OR 2.2 [95% CI = 1.5–3.2], *p* = 0.0088). However, odds ratio for IgG3 in pre-treatment was not statistically significant.

IFNγ (OR 4.2 [95%CI 1.7–12.5], *p* < 0.0001), IL-8 (OR 58.36 [95%CI 13.4–448]; *p* < 0.0001), and TNFα (OR 3.3 [95%CI 1.5–7.9]; *p* < 0.0001) were associated with HBeAg-positive patients in the pre-treatment. IFNγ (OR 2.0 [95%CI 1.1–3.6], *p* < 0.0001), and IL-8 (OR 2.9 [95%CI 1.9–4.5], *p* < 0.0001) were associated with post-treatment samples of HBeAg-positive patients. Association between IFNα and VR patients were detected, ORs of 2.3 ([95%CI 1.7–3.1], p < 0.0001) in pretreatment samples. At pre-treatment, granzyme (OR 9.8 [95%CI 3.9–30.24], *p* < 0.0001), TRAIL (OR 5.1 [95%CI 2.0–15.35], *p* = 0.0024), and IL-8 (OR 4.9 [95%CI 1.4–20.5], *p* = 0.041), were significantly associated with the SC patients. Granzyme and TRAIL was closely associated with SC patients in post-treatment samples with ORs of 2.52 [95%CI 1.6–4.0], *p* < 0.0001) and 2.6 [95%CI 1.4–5.0, *p* = 0.027), respectively. Association between IL-8 (OR 1.3), IL-6 (OR 1.4), IL-2 (OR 1.7), and IL-10 (OR 1.5) and post-treatment samples of SC patients was detected but these were not statistically significant. Multivariate Cox regression analysis showed similar hazard ratios between the VR and SC groups in baseline HBV DNA titer, baseline ALT level, age, sex, and age (Supplemental Table S1). IgG1 and C1q and IgG1, IgG3, and C1q were significant variables related to the VR or SC groups, respectively.

## Discussion

B cells are involved in antibody production and humoral immunity where seroconversion of HBsAg is associated with the functional cure for CHB patients^11,12^. B cells are more activated in CHB compared to healthy donors^21^. In this study, we investigated the relationship between natural antibody IgG and outcomes of ETV therapy in CHB patients. B1 cell-derived natural antibodies play a critical homeostatic role in clearing apoptotic debris and pathogens and invoke activation of the immune pathway leading to lysis of enveloped virus particles long before the adaptive immune response is activated^16–18^. Complement can also induce neutralization of pathogens, regulation of inflammatory responses, and enhancement of the adaptive immune response^25–27^. We found that high pretreatment and post-treatment levels of natural antibody IgG1–4, C3, and/or C1q were significantly associated with HBeAg-positive and seroconverters (SC) (Fig. 8). These results suggest that natural antibody IgG and C3 or C1q are closely related to ETV treatment outcomes in CHB patients.

We observed that natural antibody IgG1–3 and C3 levels were higher before and after treatment in HBeAg-positive patients than those in HBeAg-negative patients (Fig. 2). HBeAg-negative patients tended to be older than HBeAg-positive patients, but the difference was not significant (Table 1). Previous studies showed that immunoglobulin-encoding genes were highly up-regulated during the HBeAg-positive immune active phase and memory B cells have enhanced differentiation into immunoglobulin-producing cells in CHB patients^20,21^. In another study, serum levels of agalactosylated IgG1 (Ag-specific Ab) were associated with the severity of liver inflammation and damage but favored treatment responses in HBeAg-positive patients^24^. B cells provided antiviral defense through production of inflammatory cytokines^28^. We found that serum levels of IFNα, IFNγ, TNFα, IL-6, IL-8, and granzyme were highly elevated in HBeAg-positive patients (Fig. 5). IL-6 and IL-8 are expressed under inflammatory conditions against viral infections^29,30^. IL-6, B cell stimulation factor 2, can increase IgM and IgG secretion in either freshly stimulated B cells or immortalized cells^31^. IFNγ and TNFα produced by T cells reduced HBV persistence by the cytokine-mediated noncytolytic process^32^. These observations suggest that the higher baseline levels of natural antibody, complement C3, and inflammatory cytokines in the HBeAg-positive patients may have a role in the treatment responses of HBeAg-positive patients. Levels between pre-and post-treatment levels of natural antibody, complement C3, and inflammatory cytokines in the HBeAg-positive patients were not much changed, suggesting that host immune regulation by natural antibody and complement may be a steady-stage process during chronic hepatitis B. In contrast, natural antibody IgG1-3 and C3 levels were lower in the HBeAg-negative patients but virological response rate at 1 year after ETV treatment was higher in these patients (Table 2). These results may be related to lower levels of baseline HBV DNA in the HBeAg-negative patients rather than the natural antibody levels. One IgG subtype, IgG2 was significantly associated with HBeAg-positive patients in pre- and post-treatment samples (Fig. 2). IgG2 can activate the complement system only in the presence of high antigen concentration^18,33^. Circulating serum HBsAg is persistently present during chronic HBV infection^34^. It is possible that high levels of IgG2 produced by circulating HBsAg may involve complement activation and opsonization of viral antigen to reduce HBV replication in HBeAg-positive patients.

Nucleoside analogs (NA) are the treatment of choice for CHB patients^8,11^. NA blocks reverse transcriptase, which inhibits HBV DNA synthesis, but is less effective at removing cccDNA or HBsAg^35,36^. Decline of serum HBsAg levels was significantly associated with HBeAg loss in HBeAg-positive patients with elevation of ALT activity by either peginterferon or ETV treatment^35^. However, in HBeAg-negative patients, HBsAg reduction was associated with peginterferon treatment but not ETV treatment^35^, indicating that a preexisting host immune response is required for HBsAg loss for CHB patients with ETV treatment. We observed that pre- and post-treatment levels of natural antibodies were associated with HBeAg-positive patients but not association with the VR patients, suggesting that natural antibodies may be an independent factor to differentiate virological response at 1 year by ETV treatment (Fig. S2).

High baseline levels of IgG2-4 and C1q, and ALT activity were closely associated with SC patients (Fig. 8). In addition, SC patients had high levels of IL-6, granzyme, and TRAIL levels (Fig. 6). B1-cell derived natural antibody IgG promotes different immune responses by interacting with innate immune cells to remove infected cells such as antibody-dependent cellular cytotoxicity, antibody-dependent cellular phagocytosis, and complement-dependent cytotoxicity^16^ and these immune responses have been reported in CHB patients^37–39^. IgG was found in the plasma membrane of hepatocytes and was associated with increased susceptibility to in vitro cytotoxicity by NK cells in CHB^37^. TRAIL-expressing NK cells were found to be enriched in the liver of CHB patients^40^. These studies suggest natural antibody-induced nonspecific immunity could be one of the pathogenic pathways participating in clearance of viral particles during chronic HBV infection. This is supported by our findings on the association of high levels of natural antibody IgG2-4 C1q, granzyme, and TRAIL with pre- and posttreatment samples of SC patients.

Polyreactive natural antibodies are used as biomarkers for patients with Alzheimer’s and Parkinson’s diseases^41^ and chronic rejection in kidney transplant^42^. Moreover, a natural antibody drug (rHIgM22) was developed for a possible treatment for multiple sclerosis (MS)^43^. In this study, observations on the association of natural antibodies with HBeAg-positive and seroconversion patients in pretreatment and posttreatment samples suggest natural antibodies could serve as biomarkers for management of CHB patients or even in therapeutics. Current treatments, including nucleotide analogues or peg-IFN for CHB, do not induce sustained seroconversion of HBsAg in PVR patients with long duration of treatment^44^. Rather than T cell lymphocytes, studies have shown important immunoregulatory roles for B cells in controlling HBV infection. Treatment of the B cell-depleting drug rituximab induced HBV reactivation in B cell lymphoma patients^45^ and in vivo depletion of B cells using rituximab led to the development of a severe form of cholangitis in mice^46^. Taken together, polyreactive natural antibodies could be considered as a therapeutic approach to restore or increase host immunity for a functional cure of CBH patients.

Limitations of this study include the patient population which was retrospective, using a nonrandomized design, and patients who are mainly infected with genotype C. Natural antibody levels were analyzed only in serum samples of CHB patients. It is possible that circulating natural antibodies could originate from other tissues than the liver in CHB patients. Further studies need to be done to recognize B1 cells and natural antibodies at the cellular level.

In summary, our study confirms previous observations that the presence of preexisting host immunity against chronic hepatitis B is important and closely related to outcome of ETV treatment. HBeAg-positive patients showed higher levels of natural antibody IgG, complement C3, inflammatory cytokines in pretreatment and posttreatment samples than in HBeAg-negative patients. In pretreatment and posttreatment, natural antibodies were closely associated with SC patients but not with VR patients. Natural antibody levels could be useful as biomarkers and possibly therapeutics for management of CHB patients.

## Materials and methods

### Study population

All participants in this study provided written informed consent. The collection of samples used in this study was approved by the Institutional Review Board of Yonsei University College of Medicine, South Korea (IRB No. 3-2018-0358). All methods were performed in accordance with the relevant guidelines and conducted according to the ethical standards laid down in the Declaration of Helsinki. A total of 126 treatment-naïve patients with CHB, who underwent ETV treatment between January 2010 and January 2013, in South Korea were enrolled in the study. Duration of the ETV treatment was from 22 to 87 months. Serum specimens were collected prior to ETV treatment as pre-treatment (baseline) and then at 5 to 7 different time points every 6 months after ETV therapy. The baseline characteristics of all patients used in this study are summarized in Table 1. Six-hundred eighty-nine pretreatment and post-treatment serum samples, ranging from a follow-up period of 1 to 87 months, were obtained from the patients with different therapy outcomes. As controls, 24 normal human serum samples that were negative for all markers of infection for hepatitis A, hepatitis B, and hepatitis C viruses were commercially acquired from Zeptometrix (Buffalo, NY).

### HBV DNA and serological assessment

Serum HBV DNA levels were quantified with the Roche COBAS Amplicor PCR assay (Roche Molecular Systems, Branchburg, NJ, USA) with the limit of detection of 116 HBV copies/ml. Levels of serum hepatitis B e antigen (HBeAg), and antibodies to HBeAg (anti-HBe) were determined by the ARCHITECT HBeAg chemiluminescence immunoassays (Abbott Laboratories, North Chicago, IL, USA). Alanine aminotransferase (ALT) activity was analyzed by the Beckman Coulter Chemistry Analyzer AU5800 (Beckman Coulter, Brea, CA, USA). Virological response (VR) was defined as undetectable HBV DNA by quantitative PCR assay as described above (< 116 copies/ml) at 1 year of ETV therapy. The follow-up period was calculated from the date of ETV treatment initiation to the date of the event or the last date of follow-up. Partial virological response (PVR) was defined as a decrease in HBV DNA of more than 1 log_10_ copies/ml (tenfold) but detectable HBV DNA at 1 year of ETV therapy. HBeAg seroconversion was defined as the loss of HBeAg and detection of anti-HBe antibody in a patient who was previously HBeAg positive and anti-HBe antibody negative during total follow-up period.

### Quantification of IgG subtypes concentration

IgG subclasses (IgG1–IgG4) in patients before and after ETV therapy were quantified by ELISA using Human IgG Subclass Profile Kit (Life Technologies Corporation, Cat# 991000) according to the manufacturer’s instruction. Briefly, 50 µL of serum samples diluted at 1:2000 for IgG1 and 1:1000 for IgG2-4 were incubated for 30 min at room temperature (18–25 °C) with anti-human IgG1–IgG4 subclass-specific antibodies, respectively. Plates were washed three times in wash buffer and peroxidase anti-human IgG solution was added and incubated for 30 min at room temperature. After three washes, the samples were developed using 3,3’,5,5’ tetramethylbenzidine and the optical density of the plate was measured at 450 nm. All samples were tested in duplicate.

### Quantification of complement C3 and C1q

Levels of complement C3 and C1q in patient’s serum before and after ETV therapy were analyzed using a Human Complement C3 ELISA kit (Abcam, Cat# ab108822) and Human Complement C1q ELISA kit (Abcam, Cat# ab170246) following the manufacturer’s instructions. Briefly, for C3 levels, 25 μl of serum samples diluted at 1:800 was mixed with the same amount of 1X biotinylated anti-C3 antibody and incubated for 2 h at the room temperature. For analyzing C1q levels, 50 μL of serum samples diluted at 1:100,000 were added to the plate and incubated for 2 h at the room temperature. After washing the plate three times, 50 μl of 1X biotinylated anti-C1q antibody were added and incubated for 1 h at room temperature. For both C3 and C1q analysis, 1X streptavidin-peroxidase conjugate was added after washing the plate and incubated for 30 min at room temperature. The samples were developed using chromogen substrate and the optical density of the plate was measured at 450 nm. Each sample was tested in duplicate.

### Host gene expression

Quantitative assessment of 9 cytokines (IL-8, Granzyme, IFNα, IFNγ, IL-10, IL-2, IL-6, TNFα, and TRAIL) in patients’ serum specimens before and after ETV therapy was performed using the Magnetic Luminex Performance Assay analyte-specific kit, (R&D systems, Minneapolis, MN) according to the manufacturer’s recommendations. Briefly, 50 μl samples were mixed with 50 μl of cytokine-specific antibody-linked magnetic beads on 96-well plates and incubated at room temperature for 2 h. Room temperature incubation steps were performed on an orbital shaker at 500 rpm. Plates were washed three times with wash buffer using a magnetic device designed to accommodate a microplate, and then incubated with biotinylated detection antibody for 1 h at room temperature on an orbital shaker at 500 rpm. Samples were washed three times as described above and resuspended in streptavidin-PE. After incubation for 30 min at room temperature on an orbital shaker at 500 rpm, and the samples were washed and resuspended in wash buffer before reading. Each sample was tested in duplicate wells. Plates were read using a Bio-Plex MAGPIX multiplex (Bio-Rad) with a lower bound of 50 beads per sample per cytokine. Each sample was measured in duplicate. xPONENT 3.1 software (Luminex Corp.) was used for data acquisition on the Bio-Plex MAGPIX multiplex (Bio-Rad). Standard wells provided by the kit were tested, and 6- or 7-point standard curves were generated with a 5PL (5-parameter logistic regression) algorithm for calculation of each cytokine level.

### Patient groups

The levels of the natural antibody subtypes (IgG1-4), complement (C3 and C1q), and cytokines (IL-8, Granzyme, IFNα, IFNγ, IL-10, IL-2, IL-6, TNFα, and TRAIL) were compared in three patient groups (Fig. 1). The first comparison group was HBeAg positive patients vs. HBeAg negative patients before ETV therapy, the second comparison group was patients with virological response (VR) vs. partial virological response (PVR), and the third comparison group was the patients with HBeAg seroconverters (SC) vs. non HBeAg seroconverters (non-SC).

### Statistical analysis

Data and statistical analyses were performed in Prism 9.3.1 (GraphPad, San Diego, CA). A *p* value ≤ 0.05, (2-tailed) was considered statistically significant. Levels of IgG subtypes (IgG1–4), C3, C1q, IL-8, granzyme, IFNα, IFNγ, IL-10, IL-2, IL-6, TNFα, and TRAIL had skewed distributions and were used after removing the outliers from nonlinear regression using the ROUT method, which is based on the False Discovery Rate (FDR), in all analyses^47^. Shaded areas in the scatter graphs indicate 95% confidence intervals (CI) around the regression lines. Medians of expression levels of IgG1–4, C3, C1q and IL-8, granzyme, IFNα, IFNγ, IL-10, IL-2, IL-6, TNFα, and TRAIL between the three patient groups (HBeAg-positive vs. HBeAg-negative; VR vs. PVR; SC vs. non-SC) were compared using nonparametric Wilcox matched-pair signed rank test. Odds ratios were calculated to analyze the associations of natural antibodies, complement, or inflammatory cytokines in the three patient groups. Simple logistic regression models were fitted for the outcome variables; HBeAg-positive vs. HBeAg-negative; VR vs. PVR; SC vs. non-SC. All models were analyzed using a univariate model, where it described the association of each individual marker (natural antibodies, complement, or inflammatory cytokines) with treatment outcome without adjusting for age, gender, ALT levels or HBV DNA levels. Forest plots were used to visually display the association between analytes and outcome variables. Multivariable Cox proportional hazards analysis was performed to analyze the relationship between levels of serum markers (IgG1–4, C3, and C1q), cytokines markers in different periods and treatment outcomes (VR and SC) with adjusting for clinical characteristics (baseline HBV DNA titer, sex, age, HBeAg status, baseline ALT level). Results of the Cox regression analysis was described by means of hazard ratio (HR) together with 95% confidence intervals. Z- and p-values were based on the Wald test using the Prism Version 9.3.1 (GraphPad, San Diego, CA).

## Supplementary Information

Supplementary Figure S1.

Supplementary Figure S2.

Supplementary Figure S3.

Supplementary Table S1.

Supplementary Legends.
