# Supplementary figures and images for: Natural antibody IgG levels are associated with HBeAg-positivity and seroconversion in chronic hepatitis B patients treated with entecavir
Source: Sci Rep. 2022 Mar 14;12:4382. doi: 10.1038/s41598-022-08457-w (PMC8921218; doi:10.1038/s41598-022-08457-w)

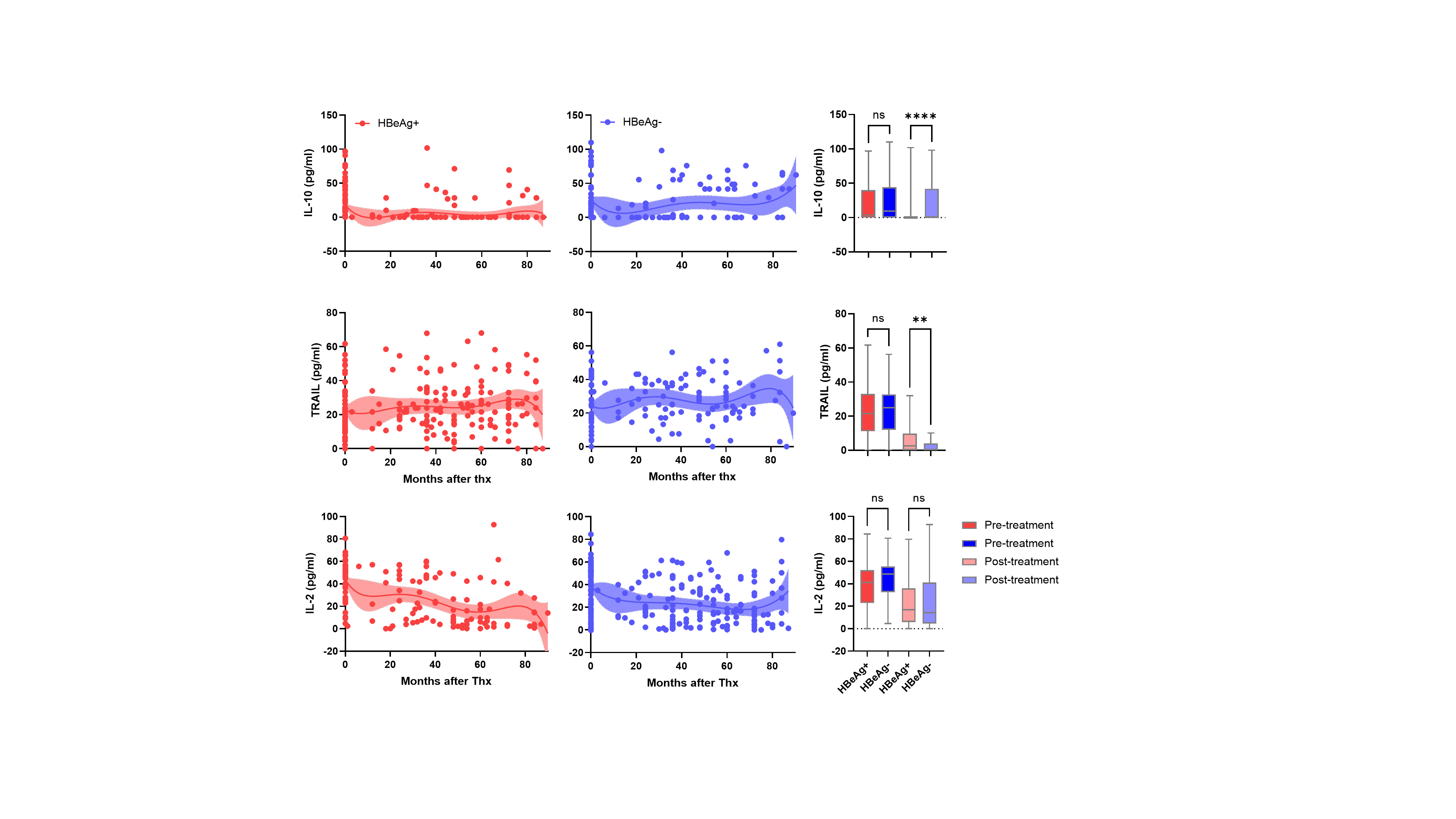

Supplement: Supplementary file 1 — Supplementary Figure S1. [file 41598_2022_8457_MOESM1_ESM.tif]

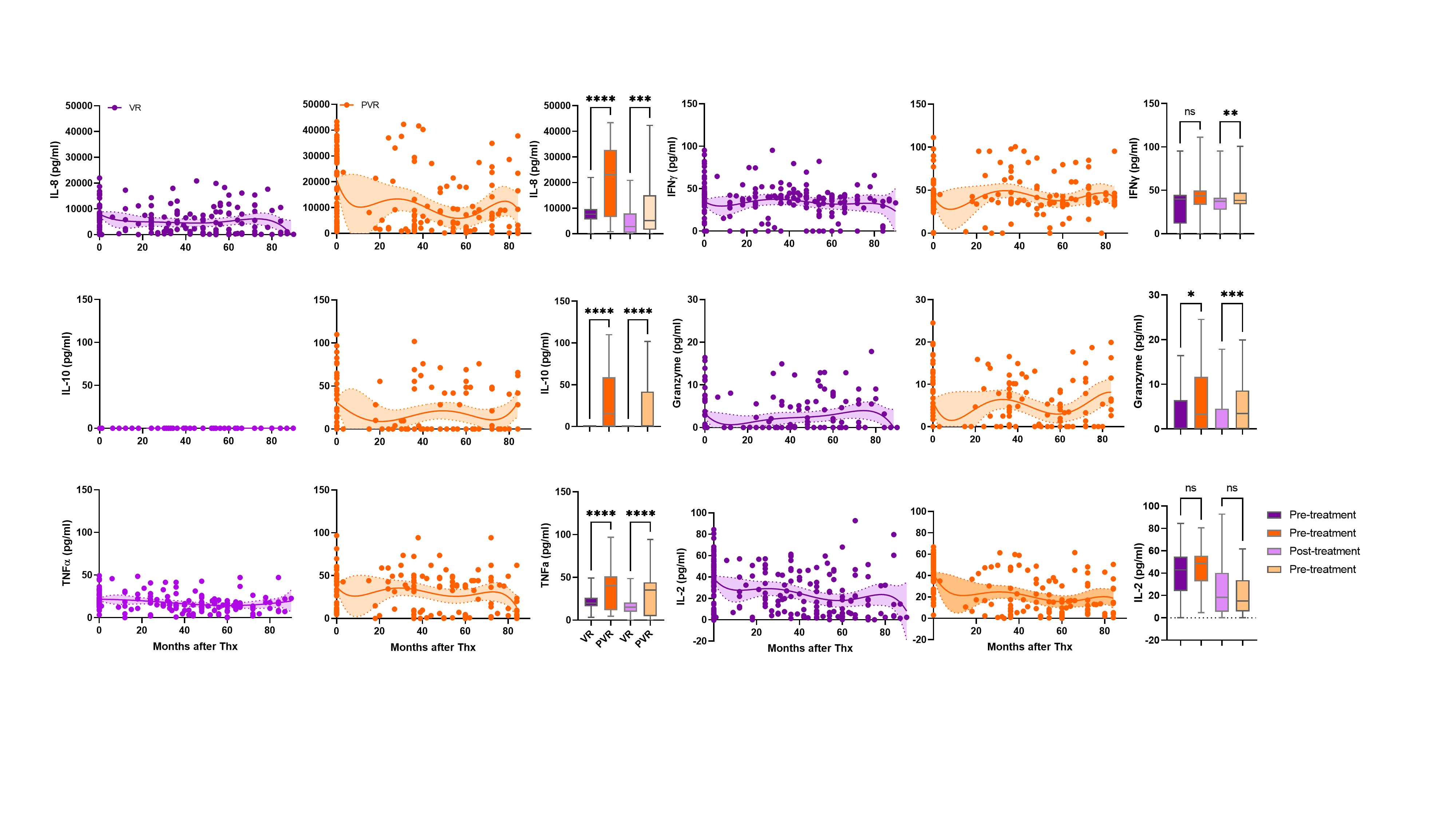

Supplement: Supplementary file 2 — Supplementary Figure S2. [file 41598_2022_8457_MOESM2_ESM.tif]

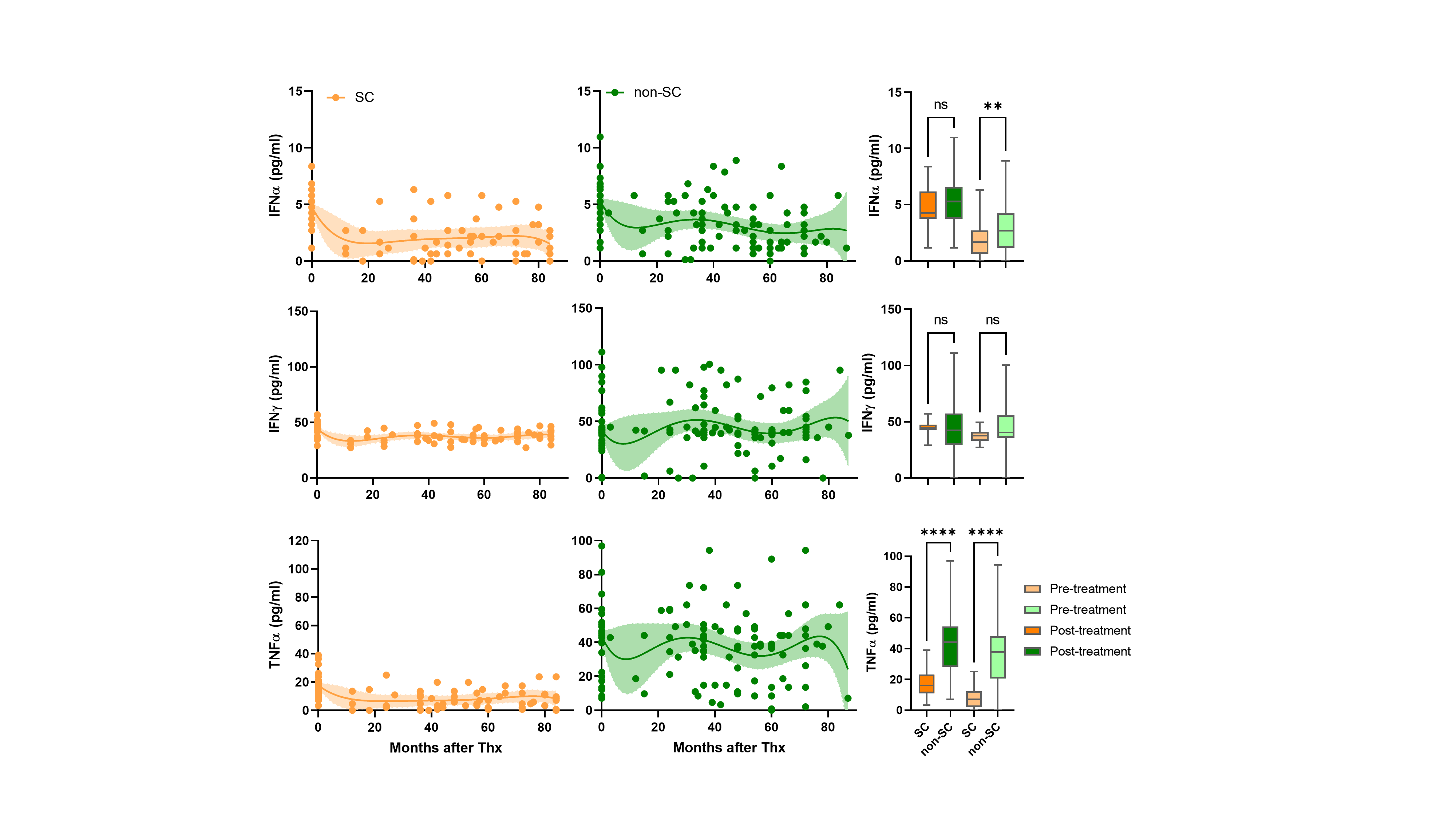

Supplement: Supplementary file 3 — Supplementary Figure S3. [file 41598_2022_8457_MOESM3_ESM.tif]
