# Supplementary material for: Natural antibody IgG levels are associated with HBeAg-positivity and seroconversion in chronic hepatitis B patients treated with entecavir
Source: Sci Rep. 2022 Mar 14;12:4382. doi: 10.1038/s41598-022-08457-w (PMC8921218; doi:10.1038/s41598-022-08457-w)
Supplement: Supplementary file 4 — Supplementary Table S1. [file 41598_2022_8457_MOESM4_ESM.docx]

Supplemental Table 1. Relationship between levels of serum markers (IgG1-4, C3, and C1q, cytokines markers and treatment outcomes (VR and SC) using multivariable Cox proportional hazards analysis.

|  | VR | | | | | SC | | | |
| --- | --- | --- | --- | --- | --- | --- | --- | --- | --- |
| Variable | | HR | 95% CI | Z value | P value | HR | 95% CI | Z value | P value |
| Baseline HBV titer | | 1 | 1.000 to 1.000 | 0.5117 | 0.6088 | 1 | 1.000 to 1.000 | 0.0699 | 0.9443 |
| Baseline ALT | | 1 | 0.9987 to 1.002 | 0.4204 | 0.6742 | 0.9991 | 0.9971 to 1.001 | 0.902 | 0.367 |
| age | | 0.9854 | 0.9757 to 0.9931 | 3.27 | 0.0011 | 1.011 | 0.9877 to 1.035 | 0.9481 | 0.3431 |
| Male Sex | | 0.7659 | 0.5147 to 1.156 | 1.295 | 0.1955 | 0.9752 | 0.5360 to 1.844 | 0.08017 | 0.9361 |
| HBeAg positive | | 0.4376 | 0.2085 to 0.9112 | 2.199 | 0.0278 | 11212 | 156.3 to 803300 | 0.000229 | 0.9998 |
| IgG 1 | | 1 | 1.000 to 1.000 | 2.006 | 0.0448 | 1 | 1.000 to 1.000 | 2.945 | 0.0032 |
| IgG 2 | | 1 | 1.000 to 1.000 | 1.436 | 0.1511 | 1 | 0.9999 to 1.000 | 0.3411 | 0.733 |
| IgG 3 | | 1 | 0.9995 to 1.001 | 0.3867 | 0.6989 | 1.001 | 1.000 to 1.002 | 2.321 | 0.0203 |
| IgG 4 | | 1 | 0.9997 to 1.001 | 0.7443 | 0.4567 | 1 | 0.9997 to 1.001 | 0.9121 | 0.3617 |
| C1q | | 1 | 1.000 to 1.000 | 3.062 | 0.0022 | 1 | 1.000 to 1.000 | 2.166 | 0.0303 |
| C3 | | 0.9999 | 0.9998 to 1.000 | 1.87 | 0.0615 | 0.9999 | 0.9997 to 1.000 | 1.858 | 0.0632 |
| IL-8 | | 1 | 1.000 to 1.000 | 8.666 | <0.0001 | 1 | 1.000 to 1.000 | 3.324 | 0.0009 |
| granzyme | | 0.9388 | 0.9193 to 0.9576 | 6.09 | <0.0001 | 0.9495 | 0.9167 to 0.9828 | 2.934 | 0.0033 |
| IFNα | | 0.925 | 0.8798 to 0.9666 | 3.296 | 0.001 | 1.025 | 0.9882 to 1.060 | 1.353 | 0.1761 |
| IFNγ | | 0.9989 | 0.9918 to 1.005 | 0.3152 | 0.7526 | 1.006 | 0.9989 to 1.014 | 1.667 | 0.0954 |
| IL-10 | | 0.9998 | 0.9981 to 1.001 | 0.2204 | 0.8256 | 0.9956 | 0.9818 to 1.007 | 0.6749 | 0.4998 |
| IL-2 | | 1.017 | 1.006 to 1.027 | 3.166 | 0.0015 | 0.999 | 0.9901 to 1.009 | 0.2027 | 0.8393 |
| IL-6 | | 1 | 1.000 to 1.000 | 3.493 | 0.0005 | 1 | 1.000 to 1.000 | 2.465 | 0.0137 |
| TNF-α | | 0.9996 | 0.9925 to 1.006 | 0.1057 | 0.9158 | 0.9907 | 0.9823 to 0.9992 | 2.145 | 0.0319 |
| TRAIL | | 0.9971 | 0.9878 to 1.006 | 0.6247 | 0.5321 | 1.012 | 0.9880 to 1.035 | 1.017 | 0.3091 |

HR, hazard ratio. Multivariable Cox proportional-hazards model in which virological response (VR) or HBeAg seroconversion (SC) was the outcome of events in different time used to evaluate the value of each variable.
