# Supplementary material for: Natural antibody IgG levels are associated with HBeAg-positivity and seroconversion in chronic hepatitis B patients treated with entecavir
Source: Sci Rep. 2022 Mar 14;12:4382. doi: 10.1038/s41598-022-08457-w (PMC8921218; doi:10.1038/s41598-022-08457-w)
Supplement: Supplementary file 5 — Supplementary Legends. [file 41598_2022_8457_MOESM5_ESM.docx]

**Supporting Information**

**Supplemental Figure 1. Cytokine levels in HBeAg-positive patients and HBeAg-negative patients.**

Circulating IL-10, TRAIL, and IL-2 levels in HBeAg-positive patients (red dots) and HBeAg-negative patients (blue dots). Shaded areas indicate 95% confidence intervals around the regression lines. Bar graphs represent concentration of cytokines in pre-treatment (dark red), post-treatment (light red) of HBeAg-positive and pre-treatment (dark blue), post-treatment (light blue) of HBeAg-negative patients. * p≤ 0.05, ** p≤ 0.01, *** p≤ 0.001, **** p≤ 0.0001.

**Supplemental Figure 2. Levels of cytokine levels in VR patients and PVR patients.** Circulating IL-8, IL-10, TNFα, IFNγ, granzyme, and TRAIL levels in VR (gray dots) patients and PVR (black dots) patients. Shaded areas indicate 95% confidence intervals around the regression lines. Bar graphs represent concentration of each cytokine in pre-treatment (dark grey), post-treatment (light grey) of VR and pre-treatment (black), post-treatment (black) of PVR patients. * p≤ 0.05, ** p≤ 0.01, *** p≤ 0.001, **** p≤ 0.0001.

**Supplemental Figure 3. Levels of cytokine levels in SC patients and no SC patients.** Circulating IFNα, IFNγ, and TNFα in SC patients (orange dots) and no SC patients (green dots). Shaded areas indicate 95% confidence intervals around the regression lines. Bar graphs represent concentration of each cytokine in pre-treatment (dark orange), post-treatment (light orange) of SC and pre-treatment (dark green), post-treatment (light green) of non-SC patients. * p≤ 0.05, ** p≤ 0.01, *** p≤ 0.001, **** p≤ 0.0001.
